# Response of Two Crop Plants, *Zea mays* L. and *Solanum lycopersicum* L., to Diclofenac and Naproxen

**DOI:** 10.3390/ijms22168856

**Published:** 2021-08-17

**Authors:** Agnieszka Siemieniuk, Michał Ludynia, Małgorzata Rudnicka

**Affiliations:** Faculty of Natural Sciences, Institute of Biology, Biotechnology and Environmental Protection, University of Silesia in Katowice, Jagiellońska 28, 40-032 Katowice, Poland; michal.ludynia@us.edu.pl (M.L.); malgorzata.rudnicka@us.edu.pl (M.R.)

**Keywords:** nonsteroidal anti-inflammatory drugs (NSAIDs), diclofenac (DFC), naproxen (NPX), maize, tomato

## Abstract

Among numerous contaminants, the ubiquitous occurrence of nonsteroidal anti-inflammatory drugs (NSAIDs) in the environment and their plausible harmful impact on nontarget organisms have made them one of the most important areas of concern in recent years. Crop plants can also potentially be exposed to NSAIDs, since the concentration of these pharmaceuticals is constantly rising in the surface water and soil. Our goal was to evaluate the stress response of two crop plants, maize and tomato, to treatment with selected NSAIDs, naproxen and diclofenac. The focus of the research was on the growth response, photosynthetic efficiency, selected oxidative stress factors (such as the H_2_O_2_ level and the rate of lipid peroxidation) as well as the total phenolic content, which represents the non-enzymatic protectants against oxidative stress. The results indicate that susceptibility to the NSAIDs that were tested is dependent on the plant species. A higher sensitivity of tomato manifested in growth inhibition, a decrease in the content of the photosynthetic pigments and a reduction in the maximum quantum efficiency of PSII and the activity of PSII, which was estimated using the F_v_/F_m_ and F_v_/F_0_ ratios. Based on the growth results, it was also possible to reveal that diclofenac had a more toxic effect on tomato. In contrast to tomato, in maize, neither the content of the photosynthetic pigments nor growth appeared to be affected by DFC and NPX. However, both drugs significantly decreased in maize F_v_ and F_m_, which are particularly sensitive to stress. A higher H_2_O_2_ concentration accompanied, in most cases, increasing lipid peroxidation, indicating that oxidative stress occurred in response to the selected NSAIDs in the plant species that were studied. The higher phenolic content of the plants after NSAIDs treatment may, in turn, indicate the activation of defense mechanisms in response to the oxidative stress that is triggered by these drugs.

## 1. Introduction

The pharmaceutical industry, which has been dynamically expanding in recent decades, has led to environmental pollution by drugs. Among the many pharmaceuticals, non-steroidal anti-inflammatory drugs (NSAIDs), which are used in both veterinary and human medicine, are a group of the most common pharmaceutical contaminants. Although they differ in terms of their chemical structure, their mode of action as pain-relieving agents is quite similar [1]. All of them inhibit cyclooxygenases (COX), which are involved in the synthesis of the prostaglandins responsible for inflammation [2]. As a consequence of their wide availability (in many countries they are sold as over the counter drugs) and the lack of efficient systems of NSAIDs elimination, they also became ubiquitous in the environment, in particular, in municipal wastewater. Their occurrence in surface water as well as their plausible impact on aquatic organisms have made them one of the most important areas of concern in recent years.

An analysis of the ecotoxicity of NSAIDs (diclofenac, ibuprofen and acetaminophen) on *Daphnia magna* revealed their toxic impact on reproduction, for which diclofenac had the highest toxicity [3].

Among the aquatic vertebrates, numerous studies on diclofenac toxicity on fish have revealed its negative impact on the liver, kidneys, gills or thyroid [4,5]. The poisoning effect of NSAIDs has also been revealed in terrestrial animals such as scavenging birds [6,7,8]. Recently, the first case of vulture poisoning by diclofenac was reported in Europe (Spain), and this is indicative of the insufficient regulations that have been implemented to prevent the intoxication of avian scavengers by NSAIDs [9].

It is worth noting that this drug toxicity in non-target predatory and scavenger animals is caused by the accumulation of the drugs in consecutive levels of the trophic pyramids, with algae as well as aquatic and terrestrial plants as the primary producers. Hence, recently, the impact of anti-inflammatory drugs on aquatic plants and algae has also been extensively studied [2,10,11]. The early results suggest that NSAIDs can also have an impact on the growth and development of terrestrial plants. However, the scarcity of data does not permit the effect of NSAIDs to be clearly defined, which leaves this problem without a satisfactory explanation.

Meanwhile, NSAIDs are present in soils that have been contaminated by sewage sludge and wastewater in concentrations up to low mg kg^−1^, which has a potential impact on crop plants [12]. For instance, radish (*Raphanus sativus* L.) and lettuce (*Lactuca sativa* L.) that had been exposed to selected NSAIDs had significant changes in their photosynthetic properties and morphological characteristics [12]. Maize plants and grains that had been treated with acetaminophen also responded with decreases in both their dry weight and the weight of their grains [13]. Prolonged irrigation of soil (up to three years) by wastewater that contained diclofenac resulted in its accumulation in tomato fruits [14]. In turn, tomato plants that had been irrigated with a nutrient solution that contained diclofenac for three months had changes in the quality of their fruits, although no diclofenac was detected in the fruits. These results could suggest that the transformation products of diclofenac are responsible for changes in the quality of fruits [15]. Moreover, recently published studies on leafy vegetables, orache (*Atriplex patula* L.), spinach (*Spinacia oleracea* L.) and lettuce (*Lactuca sativa* L.), that had been exposed to diclofenac, naproxen and ibuprofen showed a decrease in their photosynthetic pigments, polyphenols and flavonoids in a dose-dependent manner, as well as damage to the chloroplasts and cell wall [16]. 

In light of the water deficit caused by climate change, the use of reclaimed wastewater for irrigation seems to be an effective way to counteract such threats. Unfortunately, this comes with the risk of soil contamination with hazardous substances such as NSAIDs. Additionally, contamination of soils with these pharmaceuticals is related to their infiltration to the surface water from municipal, agricultural and industrial wastewater effluents [17,18]. As yet, no data are available on a completely effective system of NSAIDs removal. Hence, the potential impact of NSAIDs on crop plants could affect productivity and food security and could also be a potential route for the exposure of livestock and humans.

To date, in the case of crop plants, more attention has been paid to the accumulation and metabolism of NSAIDs, while their role as possible plant stressors remains insufficiently understood.

The aim of this work was to assess the stress response of two crop plant species from to different clades, maize (*Zea mays* L.) and tomato (*Solanum lycopersicum* L.), to selected NSAIDs, diclofenac and naproxen, which are used worldwide as pain-relieving agents and are present in wastewater treatment plants, as well as in the aquatic environment [19,20,21]. The selected plant species represent some of the most widely cultivated crops.

Our research focused on the growth response of plants, their photosynthetic parameters, selected oxidative stress factors (such as their H_2_O_2_ level and rate of lipid peroxidation) and their total phenolic content, which represents the non-enzymatic protectants against oxidative stress.

## 2. Results and Discussion

### 2.1. Plant Growth

Two crop plants, a monocotyledon representative, maize (*Zea mays* L. cv. Cosmo 230), and a dicot plant, tomato (*Solanum lycopersicum* L. ‘Moneymaker’), were exposed to a dose (2 mg L^−1^) of two selected nonsteroidal anti-inflammatory drugs: diclofenac (DFC) and naproxen (NPX).

Plant growth is a common factor that is used to assess potential phytotoxicity [22]. The growth response to the NSAIDs that were examined in this work clearly differed depending on the plant species that was tested.

**Figure 1:**
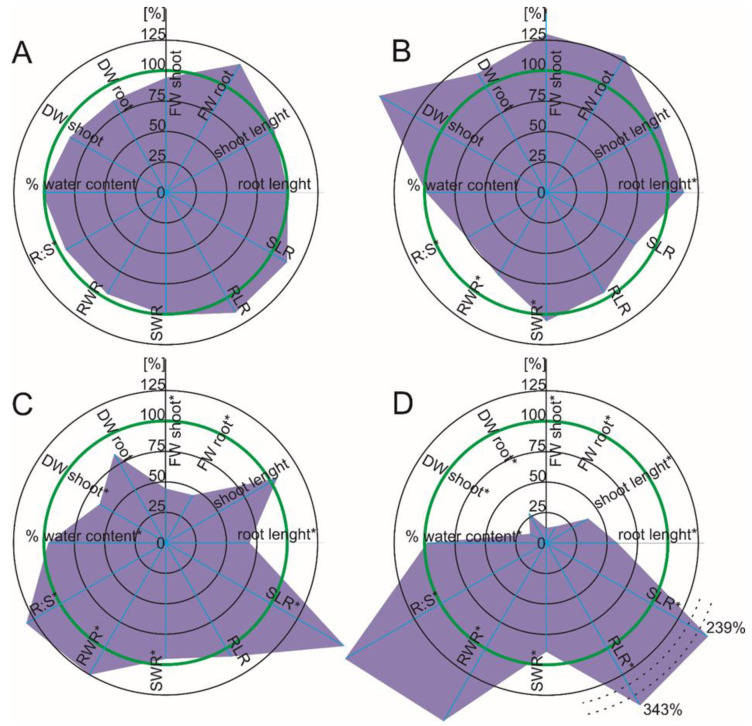
Spider plot of the selected growth parameters of the plants in response to diclofenac. The data are shown as a percentage of control; the green line indicates the control level of presented parameters. DW—dry weight, FW—fresh weight, RWR—root weight ratio, SWR—shoot weight ratio, RLR—root length ratio, SLR—shoot length ratio, R:S—root to shoot ratio, WC—water content. Values that are significantly different from the control by the unpaired Student’s *t*-test (*p* < 0.05) are marked with an asterisk. (**A**) *Zea mays* L., seven days after diclofenac was added; (**B**) *Zea mays* L., 14 days after diclofenac was added; (**C**) *Solanum lycopersicum* L., seven days after diclofenac was added; (**D**) *Solanum lycopersicum* L., seven days after diclofenac was added. In order to maintain the clarity of the D chart, a dashed line break for SLR and RLR was applied, and the percentage values were expressed in numbers.

**Figure 2:**
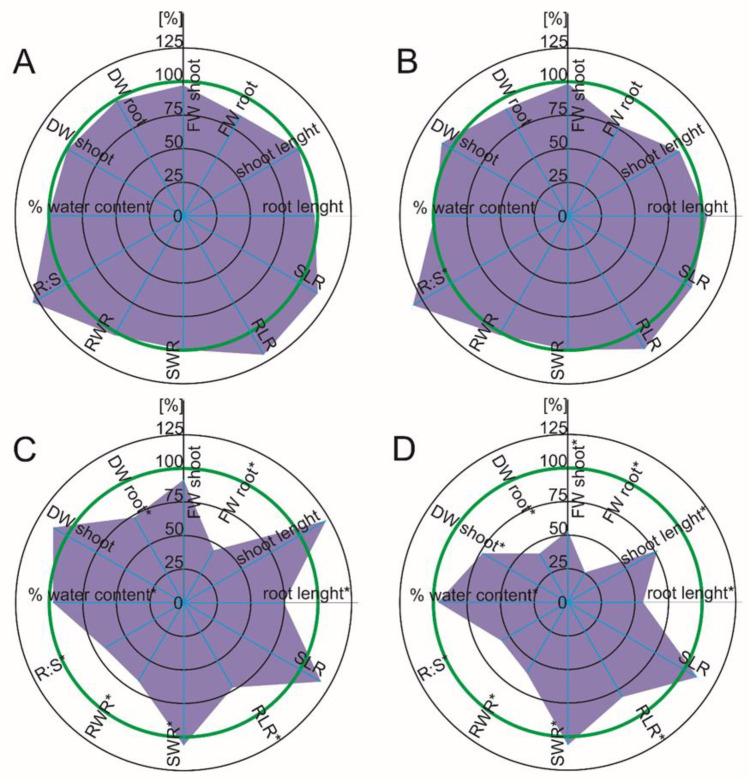
Spider plot of the selected growth parameters of the plants in response to naproxen. The data are shown as a percentage of the control; the green line indicates the control level. DW—dry weight, FW—fresh weight, RWR—root weight ratio, SWR—shoot weight ratio, RLR—root length ratio, SLR—shoot length ratio, R:S—root to shoot ratio. Values that are significantly different from the control by the unpaired Student’s *t*-test (*p* < 0.05) are marked with an asterisk. (**A**) *Zea mays* L., seven days after naproxen was added; (**B**) *Zea mays* L., 14 days after naproxen was added; (**C**) *Solanum lycopersicum* L., seven days after naproxen was added; (**D**) *Solanum lycopersicum* L., seven days after naproxen was added.

Diclofenac had no significant effect on the growth parameters of the maize, which were measured one week after the drug was added. After two weeks of exposure to DFC, there was a slight but significantly relevant root length increase (12.7%) compared to the control. Simultaneously, there was a significant decrease (above 21%) of the root weight ratio (RWR) and root to shoot ratio (R:S) (25%), which suggests biomass allocation to the aboveground parts of the plants (Figure 1A,B). Regardless of the duration of drug exposure, naproxen in the growth medium did not change the maize growth parameters, which was similar to diclofenac. An exception was a higher value of the R:S ratio, which suggests changes in the biomass proportion in favor of the roots (Figure 2A,B).

While *Zea mays* L. is almost insensitive to NPX and DFC at the concentration that was tested, the growth of *Solanum lycopersicum* L. was visibly affected by both pharmaceuticals.

When tomato was exposed to DFC, there was a significant growth inhibition in both parts of the plants, especially two weeks after diclofenac was added to the growth medium, which was manifested inter alia in both the fresh and dry mass of the plants. The decrease in growth was significantly worse with a longer duration of drug exposure (Figure 1C,D). A lower, but also significant, growth inhibition was found in tomato after NPX treatment (Figure 2C,D).

It is worth noting that the growth inhibition of tomato, which had been treated with NPX, started from the roots (root dry and fresh weight, root length). As an example, there was a 55% decrease of root FW and a 25% decrease of root length in the presence of NPX, although the growth of aboveground parts of the tomato plants did not change significantly. Two weeks after drug administration, there was a growth inhibition of the aboveground parts of the plants (an approximately 48% decrease of the shoot FW and a 44% decrease of the root length in response to NPX) (Figure 2C,D). Two weeks after the treatment with both drugs, there was a significant growth inhibition in both the roots and shoots (Figure 1C,D and Figure 2C,D). The earlier root growth inhibition, especially in response to NPX, might have been because when the roots were immersed in the medium that contain the pharmaceuticals, they were directly exposed to them. This type of contact of plant roots with contaminants may lead to their increased sensitivity, which would be manifested by the stimulation or inhibition of elongation growth, cell division or differentiation and changes in the viability of the root tissue cells [23]. In turn, the tomato plants that had been treated with DFC manifested a significant increase in RLR and SLR, with a concomitant fresh and dry weight decrease, which could indicate that the decrease in the total root and shoot area resulted from limited branching. The tomato plants also exhibited a different pattern of changes in the biomass distribution in response to both pharmaceuticals. While the plants that had been treated with DFC had higher values of R:S and RWR, which indicates a biomass allocation to roots, NPX caused a reversed trend.

In the literature, the results concerning plant growth in response to NSAIDs are contradictory. For instance, in their experiments, Sousa et al. [24] showed that the growth of tomato shoots in response to DFC (5 mg L^−1^) was significantly inhibited, while in the roots, there was only a slight decrease in their growth. An increased R:S ratio after DFC treatment, which was also reported by the authors, was comparable to the pattern in water stress, in which the allocation of the photosynthates to the roots enables them to improve water uptake and also reduce water loss due to the smaller shoot area. However, it is worth noting that the exposure of plants to DFC was much longer (five weeks) compared to our studies. Schmidt and Redshaw [12] reported, in turn, a decrease of the radish R:S ratio after DFC, which is in line with our results for maize and confirms the diversity of the responses to NSAIDs among the various plant species.

Other results have shown an increase in the primary root length of *Solanum lycopersicum* L. that was grown in a medium with DFC (5 and 10 mg L^−1^) [23]. Although the exposure time to the DFC was the same as in one of the variants in our experiment, the plants were at an earlier stage of development. This leads to the suggestion that the tomato plant sensitivity to selected NSAIDs depends on the stage of plant development. In *Zea mays* L., the growth of the primary roots was not affected by DFC, which is in line with our results that indicate that DFC had no effect on root length and mass. However, an analysis of *Zea mays* L. growth in the presence of DFC (1, 5 and 10 mg L^−1^) for 20 days indicated a root and stem decrease [23]. These data are contradictory to the results presented here, in which the maize plants were practically insensitive to both DFC and NPX.

### 2.2. Photosynthetic Pigment Content and Chlorophyll Fluorescence

**Figure 3:**
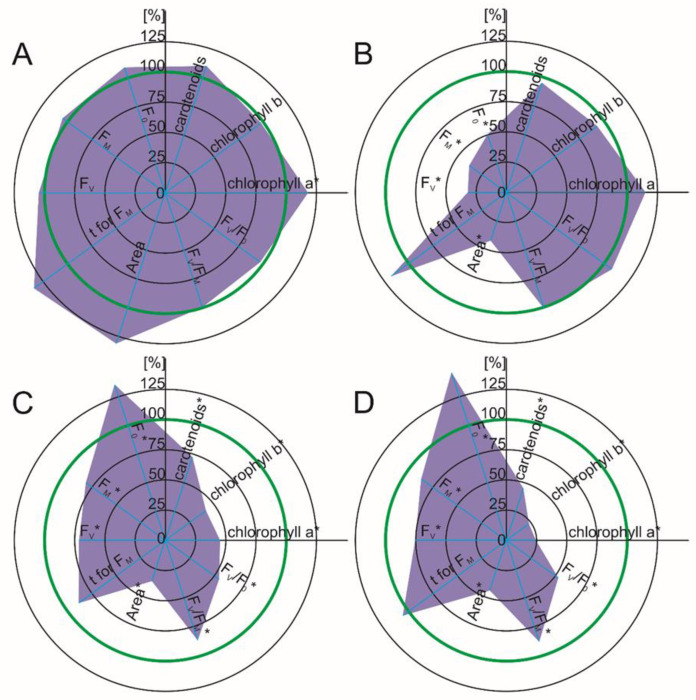
Spider plot of the selected photosynthetic parameters (photosynthetic pigments and the Chl a fluorescence parameters that characterize PSII) of the leaves in response to diclofenac. The data are shown as a percentage of the control; the green line indicates the control level. F_0_—minimal fluorescence, F_m_—maximal fluorescence, F_V_—variable fluorescence, Area—a total complementary area between the fluorescence induction curve and F_m_, F_V_/F_m_—maximum quantum efficiency of PSII after dark adaptation, F_v_/F_0_—the ratio between the rate constants of the photochemical and nonphotochemical deactivation of the excited Chl molecules, T to F_m_—time to reach maximal fluorescence. Values that are significantly different from control by the unpaired Student’s *t*-test (*p* < 0.05) are marked with an asterisk. (**A**) *Zea mays* L., seven days after diclofenac was added; (**B**) *Zea mays* L., 14 days after diclofenac was added; (**C**) *Solanum lycopersicum* L., seven days after diclofenac was added; (**D**) *Solanum lycopersicum* L., seven days after diclofenac was added.

The rate of photosynthesis is an important indicator that indicates the toxic effect of pollutants such as pharmaceuticals. It can be evaluated by parameters such as the photosynthetic pigment content of chlorophyll fluorescence. Our results revealed a clearly visible decrease in all of the photosynthetic pigments (chlorophyll a, b and carotenoids) in the tomato leaves in the response to DFC (55%, 59% and 28% decrease seven days after DFC was added and 75%, 77% and 55% decrease 14 days after DFC was added) (Figure 3C,D). While a chlorophyll deficiency leads directly to disturbances in the conversion of light into chemical energy during photosynthesis, a reduced content of carotenoids weakens the defense mechanism against oxidative stress [25]. It has been reported, in turn, that the concentration of ROS increases in plants in response to various NSAIDs [24,25,26,27,28]. Hence, a combination of these factors could enhance the effect of selected NSAIDs as environmental stressors.

**Figure 4:**
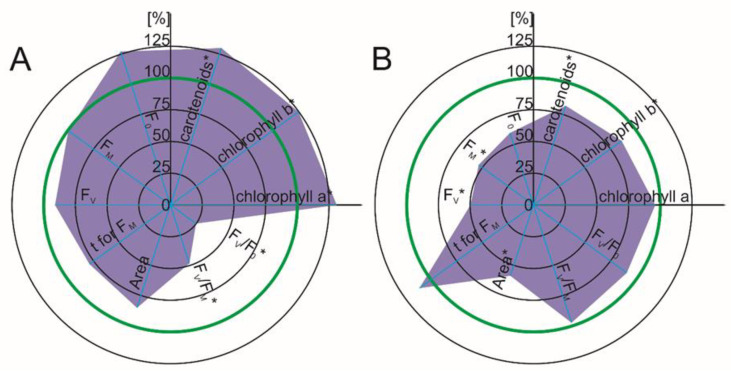

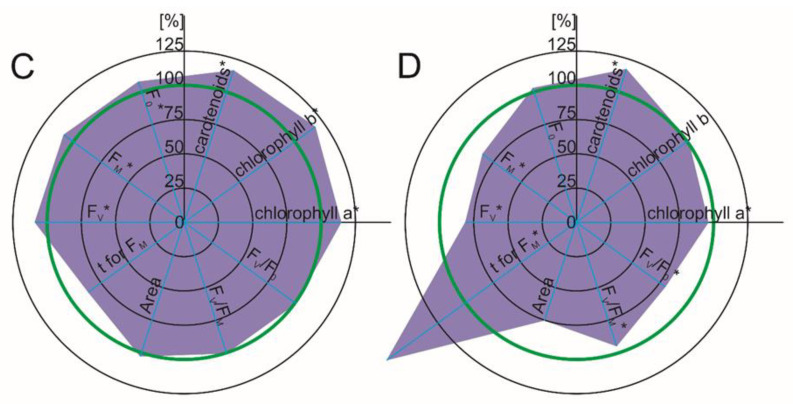
Spider plot of the selected photosynthetic parameters (the photosynthetic pigments and Chl a fluorescence parameters that characterize PSII) of the leaves in response to naproxen. The data are shown as a percentage of the control; the green line indicates the control level. F_0_—minimal fluorescence, F_m_—maximal fluorescence, F_V_—variable fluorescence, Area—a total complementary area between the fluorescence induction curve and F_m_, F_V_/F_m_—maximum quantum efficiency of PSII after dark adaptation, F_v_/F_0_—the ratio between the rate constants of the photochemical and nonphotochemical deactivation of the excited Chl molecules, T to F_m_—time to reach maximal fluorescence. Values that are significantly different from the control by the unpaired Student’s *t*-test (*p* < 0.05) are marked with an asterisk. (**A**) *Zea mays* L., seven days after naproxen was added; (**B**) *Zea mays* L., 14 days after naproxen was added; (**C**) *Solanum lycopersicum* L., seven days after naproxen was added; (**D**) *Solanum lycopersicum* L., seven days after naproxen was added.

In maize, the photosynthetic pigment concentration did not decrease significantly in response to either DFC or NPX (Figure 3A,B and Figure 4A,B). Moreover, in the presence of NPX, there was a higher concentration of the pigments in the maize leaves, which suggests that it has a lower sensitivity to NSAIDs (Figure 4A,B). The data that has been published by others differ slightly from our results. Zezulka et al. [23] showed a decrease in the chlorophyll content in maize in response to DFC at comparable concentrations. However, similar to our results, the carotenoid content did not change in response to DFC.

The literature data concerning the photosynthetic pigment content in response to NSAIDs vary among plant species, which indicates their different degrees of sensitivity to contamination by these drugs, but it may also differ depending on the concentration of the pharmaceutical.

For example, *Lemna* spp. that had been treated with low concentrations of diclofenac (20–100 µg L^−1^) had a higher concentration of total chlorophylls and carotenoids [10]. Studies that were conducted on leafy vegetables (*Atriplex patula* L., *Spinacia oleracea* L. and *Lactuca sativa* L.) revealed a decrease in the total chlorophyll in the leaves of plants that had been treated with diclofenac and naproxen [16].

In some plants species, changes in the content of the plant pigments require very high NSAID concentrations (100 mg L^−1^) [29].

Since photosynthesis is characterized by a high susceptibility to abiotic stresses, the parameters of chlorophyll fluorescence are a very useful tool for assessing changes in the photosynthetic apparatus in response to selected NSAIDs [30].

The measurements of chlorophyll fluorescence of the maize leaves showed that seven days after NPX was added to the growth medium, the F_v_/F_m_ and F_v_/F_0_ ratios were markedly lower (approximately a 52% and 75% reduction, respectively), while 14 days after the drug was added, there was a decrease in both F_v_ and Fm and Area (about a 51%, 47% and 42% decrease, respectively) (Figure 4A,B). In the tomato, seven days after the NPX was added, F_0_, F_m_ and F_v_ increased slightly (about 8%, 9% and 9%, respectively). The chlorophyll fluorescence measurements of the tomato leaves 14 days after NPX was added revealed a decrease in the F_m_, F_v_ and F_v_/F_m_ and F_v_/F_0_ ratios (about a 16%, 19%, 5% and 20% decrease, respectively) and a visible increase of T to F_m_ (71% time extension) (Figure 4C,D).

Adding diclofenac to the growth medium of *Zea mays* L. did not change the measured parameters of chlorophyll fluorescence in the shorter time frame (one week) (Figure 3A,B). Two weeks after DFC was administered, the F_0_, F_v_, Fm and Area decreased significantly (50%, 68%, 62% and 58% decrease, respectively). In response to diclofenac, the tomato leaves had lower values of F_m_, F_v_, Area, F_v_/F_m_ and F_v_/F_0_, regardless of the duration of the experiment (19%, 29%, 65%, 13% and 46% decrease, seven days after DFC was added; 14%, 25%, 57%, 13% and 48% decrease, 14 days after DFC was added). Moreover, the values of F0 were higher in both time frames of the experiment (a 35% and 46% increase) (Figure 3C,D).

The higher values of initial fluorescence, F_0_, particularly in tomato in response to DFC, could have resulted from the destruction of the PSII reaction centers, which leads to disturbances in the energy transfer from the light-harvesting complex to the PSII reaction center [31,32].

Higher F_0_ values, together with a concomitant decrease in the photosynthetic pigments, which constitute the light-harvesting complex, may be responsible for reducing the light-harvesting capacity, and therefore, there is an inefficient energy transfer from the pigments to the photosynthetic reaction centers [33]. A decrease in the maximum fluorescence (F_m_) is related to a partial reduction of the PSII electron acceptors, which is characteristic in stress conditions. Its value also depends on the chlorophyll content. Hence, a decrease in chlorophyll might be the reason for the lower F_m_ [34].

Variable fluorescence (F_v_) reflects the maximum quantum yield of PSII, and its lower value could mean a decrease in PSII activity and the thermal dissipation of excitation energy. A lower F_v_ can result from the stress factors that are responsible for damage to the thylakoid membrane [34].

The Chl fluorescence kinetics revealed that the maximum quantum efficiency of PSII and the activity of PSII, which were estimated by F_v_/F_m_ and F_v_/F_0_, were lower when tomato was treated with DFC. A decrease in F_v_/F_m_ by DFC has also been reported by other authors in *Zea mays* L. at a comparable concentration of the drug [23].

F_v_/F_m_ is a ratio that indicates a relatively low sensitivity to stress factors. A much more sensitive ratio is F_v_/F_0_ [35,36]. Only a partial photoinhibition in stress conditions results in a larger decrease in F_v_/F_0_ value in leaves [37].

Treating the plants with the selected NSAIDs also caused a decrease in the total complementary area between the fluorescence induction curve and F_m_ (Area), which indicates a pool size of reduced plastoquinone on the reducing side of PSII [38,39]. The most probable reason for this decrease is the inhibition of electron transfer from the reaction center to the quinone pool. A similar decrease has been detected, e.g., in response to heavy metals [39,40,41].

### 2.3. Oxidative Stress Factors and Polyphenols as the Antioxidant Defence System

Numerous abiotic stressors can easily disrupt the equilibrium between ROS generation and ROS scavenging by antioxidants, which leads to oxidative stress as a result of ROS accumulation. Oxidative stress can induce lipid peroxidation, damage to the nucleic acids and proteins and carbohydrate metabolism disturbances, resulting in cell dysfunction and death [42]. Hence, the level of oxidative stress in response to the selected NSAIDs was estimated by measuring its markers, like H_2_O_2_ concentration in over and underground parts of plants, as well as the level of lipid peroxidation.

**Figure 5:**
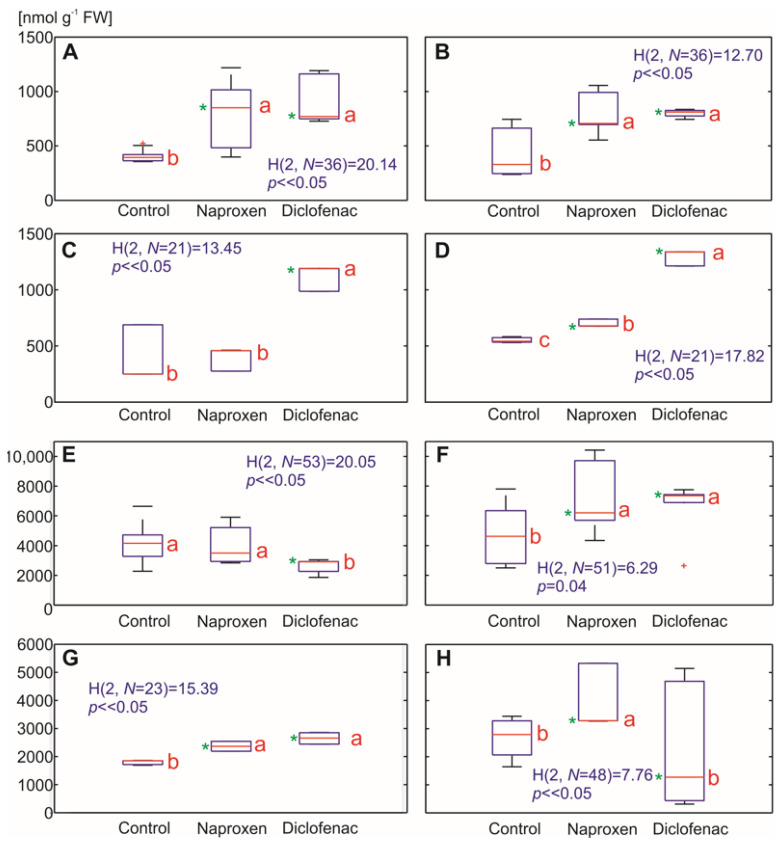
The effect of the selected NSAIDs on the hydrogen peroxide (H_2_O_2_) level in the plant tissues. Each box of the boxplots includes the median (central red line), the 25th and 75th percentiles (the edges of the box), extreme data points (whiskers) and outliers (red pluses). The statistical analysis was performed based on the Kruskal–Wallis test (the values are presented in blue). The means with the same red letters close to the median are not significantly different from each other. A significance level of 5%, where ∝ = 0.05 (critical value) and a p (probability) value that was determined from the distribution of the test statistics were used for all of the analyses. Additionally, values that were significantly different from the control using the Mann–Whitney U test (*p* < 0.05) are marked with a (green) asterisk. (**A**) *Zea mays* L. root, seven days; (**B**) *Zea mays* L. root, 14 days, root; (**C**) *Solanum lycopersicum* L. root, seven days; (**D**) *Solanum lycopersicum* L root., 14 days after drug administration; (**E**) *Zea mays* L. leaves, seven days; (**F**) *Zea mays* L. leaves, 14 days, root; (**G**) *Solanum lycopersicum* L. leaves, seven days; (**H**) *Solanum lycopersicum* L leaves., 14 days after drug administration.

Among the ROS, H_2_O_2_ is formed in plants as part of their normal cellular metabolism. It plays a signaling role in many physiological processes such as ABA-mediated stomatal closure or root gravitropism, which is regulated by auxin. Moreover, H_2_O_2_ modulates the tolerance response under various biotic and abiotic stresses [43]. Measuring the H_2_O_2_ levels in the plant tissues revealed an increase in its concentration in response to the selected NSAIDs in a medium. In the maize roots, one week after the NSAIDs were added, there was an increase in the H_2_O_2_ concentration in response to both drugs (a 90% increase for NPX and a 129% increase DFC). After two weeks, a significantly higher level of H_2_O_2_ was also observed in the maize roots that had been treated with NPX and DFC (88% and 94% increase, respectively) (Figure 5A,B). In the tomato roots, a higher level of H_2_O_2_ was especially visible in response to DFC (an approximately 152% and 132% increase one and two weeks after DFC was added) (Figure 5C,D).

In the maize leaves, there was an increase in the H_2_O_2_ concentration two weeks after treatment with both of the NSAIDs that were used in the experiment (a 48% increase for NPX and a 34% increase for DFC) (Figure 5E,F). The tomato leaves had an increased H_2_O_2_ production in response to both drugs, regardless of the duration of the experiment (from a 30% to almost a 60% increase). An exception was the H_2_O_2_ level in the tomato leaves of the plants that were grown with DFC for two weeks, for which the results were widely scattered (Figure 5G,H). This was likely because some of the plants in this variant of the experiment showed signs of dying, which could be associated with a rapid drop in the level of H_2_O_2_ production.

A higher level of H_2_O_2_ in response to DFC at a comparable concentration in maize and tomato roots has also been reported by other authors [23,24]. An increased production of ROS, in particular a hydroxyl radical, is predominantly accompanied by a higher level of lipid peroxidation. Malondialdehyde (MDA), which is one of the final products of the peroxidation of unsaturated fatty acids in the membrane lipids, affects the cell membrane properties by changing their fluidity and permeability [44]. In the presence of Cu, Fe or Mn ions as catalysts, it is possible to transform H_2_O_2_ into a hydroxyl radical [45].

**Figure 6:**
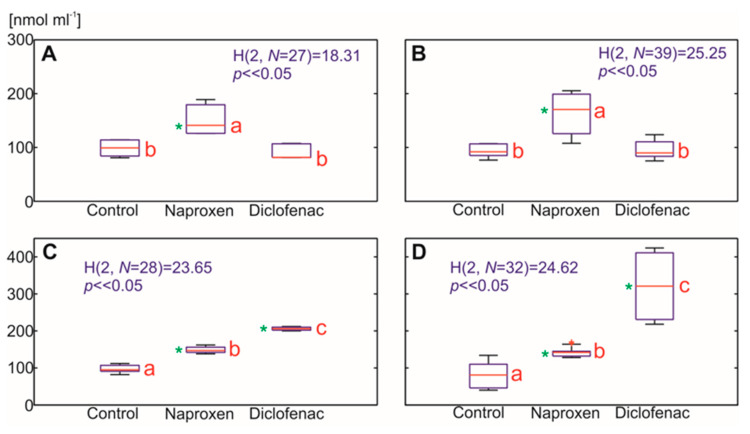

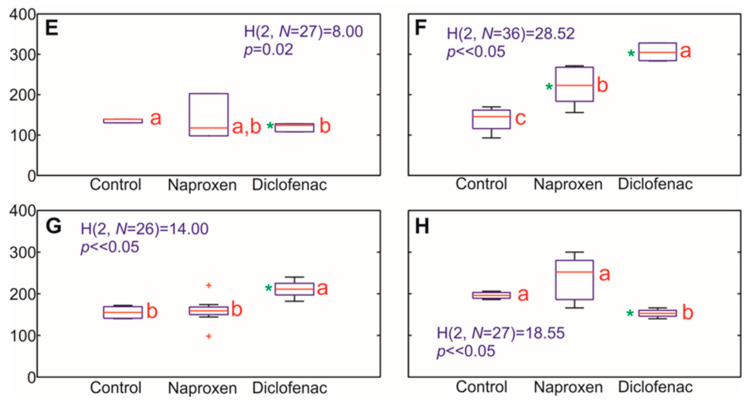
The effect of the selected NSAIDs on lipid peroxidation (MDA content) in the plant tissues. Each box of the boxplots includes the median (central red line), the 25th and 75th percentiles (the edges of the box), extreme data points (whiskers) and outliers (red pluses). The statistical analysis was performed based on the Kruskal–Wallis test (the values are presented in blue). The means with the same red letters close to the median are not significantly different from each other. A significance level of 5%, where ∝ = 0.05 (critical value) and a p (probability) value that was determined from the distribution of the test statistics were used for all of the analyses. Additionally, values that were significantly different from the control using the Mann–Whitney U test (*p* < 0.05) are marked with a (green) asterisk. (**A**) *Zea mays* L. root, seven days; (**B**) *Zea mays* L. root, 14 days, root; (**C**) *Solanum lycopersicum* L. root, seven days; (**D**) *Solanum lycopersicum* L. root, 14 days after drug administration; (**E**) *Zea mays* L. leaves, seven days; (**F**) *Zea mays* L. leaves, 14 days, root; (**G**) *Solanum lycopersicum* L. leaves, seven days; (**H**) *Solanum lycopersicum* L leaves., 14 days after drug administration.

The above-described link was confirmed by our results, especially in tomato roots, where, in most cases, the increase in the H_2_O_2_ concentration was accompanied by an increase in MDA production (from a 1.7-fold to a 4-fold increase of MDA content) (Figure 5 and Figure 6). A lower level of MDA was observed in the tomato leaves two weeks after DFC treatment, which, along with H_2_O_2_ level in this variant, could have resulted from an affected metabolism of the plants that had the symptoms of dying. In the maize roots, an increased lipid peroxidation was mainly observed after the NPX treatment (a 52% and 73% increase of the MDA content, respectively, one and two weeks after the NPX treatment), while in the leaves, lipid peroxidation increased in response to both of the NSAIDs that were tested two weeks after the drugs were administered (164% of the control for NPX and 224% of the control for DFC).

**Figure 7:**
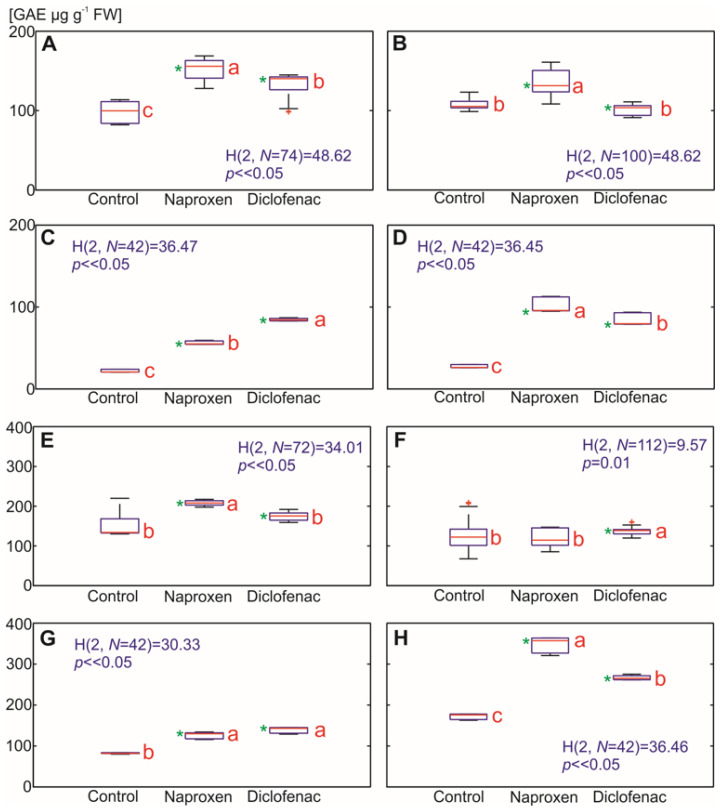
The effect of the selected NSAIDs on the total phenolic content in the plant tissues. Each box of the boxplots includes the median (central red line), the 25th and 75th percentiles (the edges of the box), extreme data points (whiskers) and outliers (red pluses). The statistical analysis was performed based on the Kruskal–Wallis test (the values are presented in blue). The means with the same red letters close to the median are not significantly different from each other. A significance level of 5%, where ∝ = 0.05 (critical value) and a p (probability) value that was determined from the distribution of the test statistics were used for all of the analyses. Additionally, values that were significantly different from the control using the Mann–Whitney U test (*p* < 0.05) are marked with a (green) asterisk. (**A**) *Zea mays* L. root, seven days; (**B**) *Zea mays* L. root, 14 days, root; (**C**) *Solanum lycopersicum* L. root, seven days; (**D**) *Solanum lycopersicum* L. root, 14 days after drug administration; (**E**) *Zea mays* L. leaves, seven days; (**F**) *Zea mays* L. leaves, 14 days, root; (**G**) *Solanum lycopersicum* L. leaves, seven days; (**H**) *Solanum lycopersicum* L. leaves, 14 days after drug administration.

Our results for lipid peroxidation are in line with the results that were published by Sousa et al. [24] on tomatoes, in which an increased MDA concentration in response to DFC was observed, with the proviso that there were more noticeable differences in the roots. An increase in the MDA content in maize and pea roots in response to DFC was also reported by [23]. Other published data reported a higher MDA content in *Pisum sativum* L. roots in response to NPX [26]. The efficiency of the plant defense mechanism against NSAIDs as a potential stressor was also determined in the form of the total polyphenol content. Polyphenols are representatives of the most important non-enzymatic protectants against oxidative stress in plants. They contain an aromatic ring with –OH or OCH_3_ substituents, which contribute to their antioxidant activity. Polyphenols are able to direct ROS scavenging and lipid peroxidation inhibition by trapping the lipid alkoxyl radical. Polyphenols also reduce the fluidity of membranes by partitioning preferentially into the hydrophobic core of the membrane and thus changing the order of lipid packing [44,46]. Although the increase in the level of polyphenols in the roots was visible in almost all variants in both plant species, the greatest differences were observed in the tomato roots (up to 3.8-fold) (Figure 7A–D). In the aboveground parts of the plants, the concentration of polyphenols also differed, depending on the plant species. An increase in the polyphenol content in response to the selected NSAIDs was more evident in the tomato (Figure 7E–H). An increase in the polyphenols in plants that had been treated with DFC and NPX is contradictory to the results that were obtained for three green leafy vegetables (*Atriplex patula* L., *Spinacia oleracea* L. and *Lactuca sativa* L.), for which there was only a moderate decrease of polyphenols in plants that had been treated with DFC and NPX at comparable concentrations (1 mg L^−1^) [16].

To sum up, there was a greater change in the H_2_O_2_ content, lipid peroxidation and polyphenol concentration in the tomato roots than in the aboveground parts of the plants in response to DFC. This probably may have resulted from their direct contact with the drugs in the medium. Other authors reported a low degree of diclofenac distribution from the roots to the aboveground parts of the plants and, as a consequence, large differences in its concentration between the root and above-ground parts of plants [28,47]. These findings, along with our results, could indicate that diclofenac induces oxidative stress in the roots and that the response in the aboveground parts of the plants is caused by a secondary oxidative stress in the roots, rather than by the direct action of DFC.

## 3. Materials and Methods

### 3.1. Plant Growth Conditions

All of the experiments were conducted using two crop plants, a monocotyledon representative, maize (*Zea mays* L. cv. Cosmo 230, Kobierzyce, Poland), and a dicot plant, tomato (*Solanum lycopersicum* L. cv. ‘Moneymaker’ from B and T World Seeds, Paguignan, France).

The seeds of *Zea mays* L. were first soaked in tap water for about 2 h, sown on wet lignin in plastic containers and then placed in growth chambers (Type MIR-533, Sanyo Electric Co., Japan) in darkness at a temperature of 27 ± 1 °C. After four days of preincubation, the seedlings were transferred to plastic nontransparent boxes (eight per box), which were filled with 2.4 L of a slightly modified Hoagland’s nutrient solution [48] (300 mL per seedling). The light-impermeable containers prevented any potential drug photodegradation and also inhibited algae growth. After three days of acclimation growth, the pharmaceuticals were added.

The seeds of *Solanum lycopersicum* L. were sown in plastic boxes on gauze that had been placed on the surface of tap water. The boxes with the germinating seeds were first kept in a room at a temperature of 20 °C in daylight. After seven days, the seedlings were gently transferred to plastic boxes that contained a Hoagland’s nutrient solution (12 seedlings per box). The plants were then cultivated in a greenhouse. Seven days after their transfer to the greenhouse (i.e., 14 days after being sown), the plants were treated with NSAIDs.

All of the plants transferred to the greenhouse were grown at 20 ± 1 °C. The light cycles for all of the plants included 14 hours of light and 10 hours of dark. The pharmaceuticals (sodium salts of naproxen and diclofenac purchased from Sigma-Aldrich USA) were added once at a final concentration of 2 mg L^−1^. During the experiment, the slight water loss from transpiration was compensated for by adding the nutrient solution without the drugs.

The durations of drug exposure experiments as well as the pharmaceutical-free control were 7 and 14 days from the moment the drug was added to the nutrient solution. All of the experiments were performed in triplicate.

### 3.2. Growth Measurements

After a set period of time of the experiment, root and shoot length was determined by a ruler, and fresh weight was estimated with use of laboratory scales.

All of the plant material was dried in a circulating air oven at 70 °C for 48 h, and then the dry weight of the aboveground and underground parts of the plants were measured. Based on these measurements, the allocation of biomass was estimated by calculating the shoot dry weight ratio (SWR, shoot DW/total DW), root dry weight ratio (RWR, root DW/total DW), shoot length ratio (SLR shoot length/total DW), root length ratio (RLR root length/total DW), root to shoot ratio (R:S root DW/shoot DW) and the tissue water content (WC) in plants, which was expressed as a percentage of the fresh weight.

### 3.3. Measurements of the Content of the Photosynthetic Pigments and Chlorophyll Fluorescence

The chlorophyll fluorescence was measured using a Pocket PEA Chlorophyll Fluorimeter, (Hansatech Instruments Ltd., King’s Lynn, UK) after 30 min of dark adaptation. The parameters that were measured included the minimum fluorescence yield (F_0_), maximum fluorescence yield (F_m_), maximum photochemical quantum yield (F_v_/F_m_), the time needed to reach the maximum fluorescence (T to F_m_) and the area above the fluorescence induction curve (Area).

The photosynthetic pigments were extracted from the leaves by homogenizing them in 80% acetone according to a slightly modified method [49]. The extract was centrifuged for 6 min in 12,300× *g*. The content of the photosynthetic pigments’ content was estimated using the spectrophotometric measurements of the samples at three wavelengths, 470, 646 and 663 nm. The pigment content was calculated according to [50].

### 3.4. H_2_O_2_ Level Determination

The hydrogen peroxide (H_2_O_2_) concentration in the plant tissues of the tested variants was determined according to [51]. Root and leaf samples of 0.1 g were homogenized in 1.5 mL of 0.1% (*w*/*v*) tri-chloroacetic acid (TCA). The homogenate was centrifuged at 12,300× *g* and 4 °C for 10 min. The reaction mixture contained 0.5 mL of the supernatant, 0.5 mL of a 0.1 M K-phosphate buffer (pH 7.0) and 1 mL of 1 M KI. After incubating the samples in the dark for 60 min, the absorbance was measured at 390 nm. The hydrogen peroxide content was calculated using a standard curve based on the absorbance of the H_2_O_2_ standards. The H_2_O_2_ concentration was expressed as nmol g^−^^1^ fresh weight (FW).

### 3.5. The Level of Lipid Peroxidation: MDA Content

The oxidative damage to the leaf and root membrane lipids was estimated as the total content of 2-thiobarbituric acid (TBA) and was expressed as the equivalent of malondialdehyde (MDA). Samples of the plant tissues (0.5 g) were homogenized in ethanol (12.5 mL), and 1 mL of the diluted sample was added to 20% TCA (-TBA solution) and 20% TCA and 0.65% TBA (+TBA solution). The mixtures were heated at 95 °C for 20 min and cooled on ice. Next, the samples were centrifuged at 12,300× *g* and 4 °C for 10 min. The absorbances of the supernatant were read at 440, 532 and 600 nm. The MDA equivalents were calculated according to [51].

### 3.6. Total Phenolic Content

Plant samples (1 g) were frozen in liquid nitrogen and homogenized and diluted in 10 mL of 80% methanol. The extract was centrifuged at 12,000× *g* and 4 °C for 15 min.

The total phenolic content of the samples was measured spectrophotometrically at 765 nm after the reaction with the Folin–Ciocalteu phenol reagent according to [52] with minor modifications. Gallic acid was used as the calibration standard. The results are given as gallic acid equivalents (GAE, µg g^−1^ FW). All of the measurements were performed in triplicate.

### 3.7. Statistical Analysis

The data were analyzed using Statistica, Version 12 (Statsoft Inc., Tulsa, OK, USA, 2013). The assumptions of normality distribution and homogeneity of variance were estimated. Based on the results of the estimation, further analyses were performed using non-parametric tests (the Mann–Whitney U test and the Kruskal Wallis test). The boxplots graphs were created using MATLAB R2009a (The Mathworks, Natick, MA, USA). Spider plots were performed with CorelDRAW^®^, Graphics Suite X7 (Corel Corporation, Ottawa, ON, Canada). For all analyses, we assumed a significance level of 0.05.

## 4. Conclusions

The study evaluated the effects of diclofenac and naproxen on two crop plants, maize and tomato. Based on the results obtained, it can be concluded that these two species differ in their susceptibility to the NSAIDs tested, since the higher stress level was observed in tomato. It should also be highlighted that the toxic effect on tomato was more evident after treatment with diclofenac. Moreover, the stress indicators measured in the present work demonstrated that tomato roots are primarily affected by diclofenac. This finding, along with earlier reports indicating the low translocation of DFC from the roots to the aboveground parts of plants, may suggest that the stress response of the shoots of the plants that had been treated with DFC may have resulted from a secondary oxidative stress in the roots that was induced by this drug. However, this newly emerged hypothesis requires verification in further research.

## Data Availability

Data is contained within the article.

## References

[1] Rao P., Knaus E.E. (2008). Evolution of Nonsteroidal Anti-Inflammatory Drugs (NSAIDs): Cyclooxygenase (COX) Inhibition and Beyond. J. Pharm. Pharm. Sci..

[2] Bácsi I., B-Béres V., Kókai Z., Gonda S., Novák Z., Nagy S.A., Vasas G. (2016). Effects of non-steroidal anti-inflammatory drugs on cyanobacteria and algae in laboratory strains and in natural algal assemblages. Environ. Pollut..

[3] Du J., Mei C.-F., Ying G.-G., Xu M.-Y. (2016). Toxicity Thresholds for Diclofenac, Acetaminophen and Ibuprofen in the Water Flea *Daphnia magna*. Bull. Environ. Contam. Toxicol..

[4] Santos L.H., Araújo A.N., Fachini A., Pena A., Delerue-Matos C., Montenegro M.C.B.S.M. (2010). Ecotoxicological aspects related to the presence of pharmaceuticals in the aquatic environment. J. Hazard. Mater..

[5] Saravanan M., Hur J.H., Arul N., Ramesh M. (2014). Toxicological effects of clofibric acid and diclofenac on plasma thyroid hormones of an Indian major carp, *Cirrhinus mrigala* during short and long-term exposures. Environ. Toxicol. Pharmacol..

[6] Hutchinson T.H., Madden J.C., Naidoo V., Walker C.H. (2014). Comparative metabolism as a key driver of wildlife species sensitivity to human and veterinary pharmaceuticals. Philos. Trans. R. Soc. B Biol. Sci..

[7] Green R.E., Newton I., Shultz S., Cunningham A.A., Gilbert M., Pain D.J., Prakash V. (2004). Diclofenac poisoning as a cause of vulture population declines across the Indian subcontinent. J. Appl. Ecol..

[8] Oaks J.L., Gilbert M., Virani M.Z., Watson R.T., Meteyer C.U., Rideout B.A., Shivaprasad H.L., Ahmed S., Iqbal Chaudhry M.J., Arshad M. (2004). Diclofenac residues as the cause of vulture population decline in Pakistan. Nature.

[9] Herrero-Villar M., Delepoulle É., Suárez-Regalado L., Solano-Manrique C., Juan-Sallés C., Iglesias-Lebrija J.J., Camarero P.R., González F., Álvarez E., Mateo R. (2021). First diclofenac intoxication in a wild avian scavenger in Europe. Sci. Total Environ..

[10] Alkimin G.D., Daniel D., Dionísio R., Soares A.M.V.M., Barata C., Nunes B. (2019). Effects of diclofenac and salicylic acid exposure on *Lemna minor*: Is time a factor?. Environ. Res..

[11] Alkimin G.D., Daniel D., Frankenbach S., Serôdio J., Soares A.M.V.M., Barata C., Nunes B. (2018). Evaluation of pharmaceutical toxic effects of non-standard endpoints on the macrophyte species *Lemna minor* and *Lemna gibba*. Sci. Total Environ..

[12] Schmidt W., Redshaw C.H. (2015). Evaluation of biological endpoints in crop plants after exposure to non-steroidal anti-inflammatory drugs (NSAIDs): Implications for phytotoxicological assessment of novel contaminants. Ecotoxicol. Environ. Saf..

[13] Hammad H.M., Zia F., Bakhat H.F., Fahad S., Ashraf M.R., Wilkerson C.J., Shah G.M., Nasim W., Khosa I., Shahid M. (2018). Uptake and toxicological effects of pharmaceutical active compounds on maize. Agric. Ecosyst. Environ..

[14] Christou A., Karaolia P., Hapeshi E., Michael C., Fatta-Kassinos D. (2017). Long-term wastewater irrigation of vegetables in real agricultural systems: Concentration of pharmaceuticals in soil, uptake and bioaccumulation in tomato fruits and human health risk assessment. Water Res..

[15] Christou A., Kyriacou M.C., Georgiadou E.C., Papamarkou R., Hapeshi E., Karaolia P., Michael C., Fotopoulos V., Fatta-Kassinos D. (2018). Uptake and bioaccumulation of three widely prescribed pharmaceutically active compounds in tomato fruits and mediated effects on fruit quality attributes. Sci. Total Environ..

[16] Opriș O., Lung I., Soran M.L., Ciorîță A., Copolovici L. (2020). Investigating the effects of non-steroidal anti-inflammatory drugs (NSAIDs) on the composition and ultrastructure of green leafy vegetables with important nutritional values. Plant Physiol. Biochem..

[17] Wojcieszyńska D., Guzik U. (2020). Naproxen in the environment: Its occurrence, toxicity to nontarget organisms and biodegradation. Appl. Microbiol. Biotechnol..

[18] Bartrons M., Peñuelas J. (2017). Pharmaceuticals and Personal-Care Products in Plants. Trends Plant Sci..

[19] Kunkel U., Radke M. (2012). Fate of pharmaceuticals in rivers: Deriving a benchmark dataset at favorable attenuation conditions. Water Res..

[20] Lacina P., Mravcová L., Vávrová M. (2013). Application of comprehensive two-dimensional gas chromatography with mass spectrometric detection for the analysis of selected drug residues in wastewater and surface water. J. Environ. Sci..

[21] Kay P., Hughes S.R., Ault J.R., Ashcroft A.E., Brown L.E. (2016). Widespread, routine occurrence of pharmaceuticals in sewage effluent, combined sewer overflows and receiving waters. Environ. Pollut..

[22] Soares C., Branco-Neves S., de Sousa A., Pereira R., Fidalgo F. (2016). Ecotoxicological relevance of nano-NiO and acetaminophen to *Hordeum vulgare* L.: Combining standardized procedures and physiological endpoints. Chemosphere.

[23] Zezulka Š., Kummerová M., Babula P., Hájková M., Oravec M. (2018). Sensitivity of physiological and biochemical endpoints in early ontogenetic stages of crops under diclofenac and paracetamol treatments. Environ. Sci. Pollut. Res..

[24] Sousa B., Lopes J., Leal A., Martins M., Soares C., Valente I.M., Rodrigues J.A., Fidalgo F., Teixeira J. (2019). Response of *Solanum lycopersicum* L. to diclofenac–Impacts on the plant’s antioxidant mechanisms. Environ. Pollut..

[25] Wang H., Jin M., Xu L., Xi H., Wang B., Du S., Liu H., Wen Y. (2020). Effects of ketoprofen on rice seedlings: Insights from photosynthesis, antioxidative stress, gene expression patterns, and integrated biomarker response analysis. Environ. Pollut..

[26] Svobodníková L., Kummerová M., Zezulka Š., Babula P., Sendecká K. (2020). Root response in *Pisum sativum* under naproxen stress: Morpho-anatomical, cytological, and biochemical traits. Chemosphere.

[27] Martins M., Sousa B., Lopes J., Soares C., Machado J., Carvalho S., Fidalgo F., Teixeira J. (2020). Diclofenac shifts the role of root glutamine synthetase and glutamate dehydrogenase for maintaining nitrogen assimilation and proline production at the expense of shoot carbon reserves in *Solanum lycopersicum* L.. Environ. Sci. Pollut. Res..

[28] Christou A., Antoniou C., Christodoulou C., Hapeshi E., Stavrou I., Michael C., Fatta-Kassinos D., Fotopoulos V. (2016). Stress-related phenomena and detoxi fi cation mechanisms induced by common pharmaceuticals in alfalfa (*Medicago sativa* L.) plants. Sci. Total Environ..

[29] Vannini A., Paoli L., Vichi M., Bačkor M., Bačkorová M., Loppi S. (2018). Toxicity of Diclofenac in the Fern *Azolla filiculoides* and the Lichen Xanthoria parietina. Bull. Environ. Contam. Toxicol..

[30] Strasser R.J., Srivastava A., Tsimilli-Michael M. (2000). The fluorescence transient as a tool to characterize and screen photosynthetic samples. Probing Photosynth. Mech. Regul. Adapt..

[31] Krause G.H., Weis E. (1984). Chlorophyll fluorescence as a tool in plant physiology-II. Interpretation of fluorescence signals. Photosynth. Res..

[32] Bolhar-Nordenkampf H.R., Long S.P., Baker N.R., Oquist G., Schreiber U., Lechner E.G. (1989). Chlorophyll Fluorescence as a Probe of the Photosynthetic Competence of Leaves in the Field: A Review of Current Instrumentation. Funct. Ecol..

[33] Yamane K., Kawasaki M., Taniguchi M., Miyake H. (2008). Correlation between chloroplast ultrastructure and chlorophyll fluorescence characteristics in the leaves of rice (*Oryza sativa* L.) grown under salinity. Plant Prod. Sci..

[34] Goltsev V.N., Kalaji H.M., Paunov M., Bąba W., Horaczek T., Mojski J., Kociel H., Allakhverdiev S.I. (2016). Variable chlorophyll fluorescence and its use for assessing physiological condition of plant photosynthetic apparatus. Russ. J. Plant Physiol..

[35] Lichtenthaler H.K., Buschmann C., Sybesma C. (1984). Photooxidative Changes in Pigment Composition and Photosynthetic Activity of Air-Polluted Spruce Needles (*Picea Abies* L.). Advances in Photosynthesis Research, Proceedings of the VIth International Congress on Photosynthesis, Brussels, Belgium, 1–6 August 1983.

[36] Babani F., Lichtenthaler H.K. (1996). Light-induced and age-dependent development of chloroplasts in etiolated barley leaves as visualized by determination of photosynthetic pigments, CO_2_ assimilation rates and different kinds of chlorophyll fluorescence ratios. J. Plant Physiol..

[37] Lichtenthaler H.K., Buschmann C., Knapp M. (2005). How to correctly determine the different chlorophyll fluorescence parameters and the chlorophyll fluorescence decrease ratio RFd of leaves with the PAM fluorometer. Photosynthetica.

[38] Kalaji H.M., Schansker G., Brestic M., Bussotti F., Calatayud A., Ferroni L., Goltsev V., Guidi L., Jajoo A., Li P. (2017). Frequently asked questions about chlorophyll fluorescence, the sequel. Photosynth. Res..

[39] Faseela P., Sinisha A.K., Brestič M., Puthur J.T. (2020). Chlorophyll a fluorescence parameters as indicators of a particular abiotistress in rice. Photosynthetica.

[40] Mathur S., Kalaji H.M., Jajoo A. (2016). Investigation of deleterious effects of chromium phytotoxicity and photosynthesis in wheat plant. Photosynthetica.

[41] Żurek G., Rybka K., Pogrzeba M., Krzyżak J., Prokopiuk K. (2014). Chlorophyll a Fluorescence in Evaluation of the Effect of Heavy Metal Soil Contamination on Perennial Grasses. PLoS ONE.

[42] Hasanuzzaman M., Bhuyan M.H.M.B., Zulfiqar F., Raza A., Mohsin S.M., Al Mahmud J., Fujita M., Fotopoulos V. (2020). Reactive oxygen species and antioxidant defense in plants under abiotic stress: Revisiting the crucial role of a universal defense regulator. Antioxidants.

[43] Neill S., Desikan R., Hancock J. (2002). Hydrogen peroxide signalling. Curr. Opin. Plant Biol..

[44] Sharma P., Jha A.B., Dubey R.S., Pessarakli M. (2012). Reactive Oxygen Species, Oxidative Damage, and Antioxidative Defense Mechanism in Plants under Stressful Conditions. J. Bot..

[45] Richards S.L., Wilkins K.A., Swarbreck S.M., Anderson A.A., Habib N., Smith A.G., McAinsh M., Davies J.M. (2015). The hydroxyl radical in plants: From seed to seed. J. Exp. Bot..

[46] Arora A., Byrem T.M., Nair M.G., Strasburg G.M. (2000). Modulation of liposomal membrane fluidity by flavonoids and isoflavonoids. Arch. Biochem. Biophys..

[47] Bartha B., Huber C., Schröder P. (2014). Uptake and metabolism of diclofenac in *Typha latifolia*—How plants cope with human pharmaceutical pollution. Plant Sci..

[48] Epstein E., Bloom A.J. (2005). Mineral Nutrition of Plants: Principles and Perspectives.

[49] Arnon D.I. (1949). Copper Enzymes in Isolated Chloroplasts. Polyphenoloxidase in *Beta Vulgaris*. Plant Physiol..

[50] Lichtenthaler H.K., Wellburn A.R. (1983). Determinations of total carotenoids and chlorophylls a and b of leaf extracts in different solvents. Biochem. Soc. Trans..

[51] Rudnicka M., Ludynia M., Karcz W. (2018). A comparison of the effects of 1,4-naphthoquinone and 2-hydroxy-1,4-naphthoquinone (lawsone) on indole-3-acetic acid (IAA)-induced growth of maize coleoptile cells. Plant Growth Regul..

[52] Djeridane A., Yousfi M., Nadjemi B., Boutassouna D., Stocker P., Vidal N. (2006). Antioxidant activity of some algerian medicinal plants extracts containing phenolic compounds. Food Chem..

